# Progression and regression of kidney disease in type 1 diabetes

**DOI:** 10.3389/fneph.2023.1282818

**Published:** 2023-12-14

**Authors:** Fanny Jansson Sigfrids, Per-Henrik Groop

**Affiliations:** ^1^ Folkhälsan Institute of Genetics, Folkhälsan Research Center, Helsinki, Finland; ^2^ Department of Nephrology, University of Helsinki and Helsinki University Hospital, Helsinki, Finland; ^3^ Research Program for Clinical and Molecular Metabolism, Faculty of Medicine, University of Helsinki, Helsinki, Finland; ^4^ Department of Diabetes, Central Clinical School, Monash University, Melbourne, VIC, Australia

**Keywords:** albuminuria, chronic kidney disease, diabetic kidney disease, diabetic complications, diagnosis, epidemiology, natural history

## Abstract

Diabetic kidney disease is distinguished by the presence of albuminuria, hypertension, declining kidney function, and a markedly elevated cardiovascular disease risk. This constellation of clinical features drives the premature mortality associated with type 1 diabetes. The first epidemiological investigations concerning type 1 diabetes-related albuminuria date back to the 1980s. The early studies found that proteinuria – largely equivalent to severe albuminuria – developed in 35 to 45% of individuals with type 1 diabetes, with the diabetes duration-specific incidence rate pattern portraying one or two peaks. Furthermore, moderate albuminuria, the first detectable sign of diabetic kidney disease, was found to nearly inexorably progress to overt kidney disease within a short span of time. Since the early reports, studies presenting more updated incidence rates have appeared, although significant limitations such as study populations that lack broad generalizability, study designs vulnerable to substantive selection bias, and constrained follow-up times have been encountered by many. Nevertheless, the most recent reports estimate that in modern times, moderate – instead of severe – albuminuria develops in one-third of individuals with type 1 diabetes; yet, a considerable part (up to 40% during the first ten years after the initial albuminuria diagnosis) progresses to more advanced stages of the disease over time. An alternative pathway to albuminuria progression is its regression, which affects up to 60% of the individuals, but notably, the relapse rate to a more advanced disease stage is high. Whether albuminuria regression translates into a decline in cardiovascular disease and premature mortality risk is an area of debate, warranting more detailed research in the future. Another unclear but alarming feature is that although the incidence of severe albuminuria has fallen since the 1930s, the decline seems to have reached a plateau after the 1980s. This stagnation may be due to the lack of kidney-protective medicines since the early 1980s, as the recent breakthroughs in type 2 diabetes have not been applicable to type 1 diabetes. Therefore, novel treatment strategies are at high priority within this patient population.

## Introduction

In 2022, a global total of 8.8 million individuals were living with type 1 diabetes (1). Encouragingly, advancements in medical care and technology have improved life expectancy for those affected by the disease, and together with the rising incidence of type 1 diabetes in many parts of the world (2), the disease prevalence will continue to rise during the following years. Indeed, it is estimated to reach over 17 million by 2040 (1). However, amidst these developments, a significant gap remains in life expectancy for those impacted by type 1 diabetes, calling for further advancements and enhancements in treatment strategies. Contemporary reports agree that the life expectancy in young adults with type 1 diabetes is around ten years shorter than that of the general population (3–5), and this disparity is explained mainly by acute and long-term co-morbidities caused by the disease (6–8). Diabetic kidney disease stands out as a particular concern due to its impact on kidney function, risk of cardiovascular disease, and, consequently, premature mortality.

The first detectable sign of kidney problems in the “traditional” course of type 1 diabetes-related kidney disease is elevated amounts of the protein albumin in the urine, termed albuminuria. Albuminuria increases as the kidney disease progresses and can, thus, also be viewed as a marker of the disease severity. The first investigations into the incidence and progression of albuminuria date back to the early 1980s. These studies were mainly small-scale single-center reports, but nevertheless laid the foundation for the clinical approach to diabetic kidney disease as we know it today. Since the early reports, many studies have encountered the same struggles regarding study design and population size. In addition, with advancements in screening and treatment strategies, the natural course of diabetic kidney disease seems to have altered over time, with albuminuria regression instead of progression standing out as a possible outcome more often than before.

This review recaps central findings from the 1980s to the 2020s on the epidemiology of type 1 diabetes-related kidney disease: its incidence, progression, and regression (summarized in **Table 1**). We focus on temporal trends and changes in the disease’s natural history. We further highlight areas of research within the topic that warrant future research.

**Table 1:** Summary of epidemiological studies relevant to this review article.

Study	Study design	Number of participants	Albuminuria determination methodology and reference limit	Main finding(s) relevant to this review
Early studies				
Andersen et al., 1983 (9)	Single-center (Steno Memorial Hospital, Denmark)	1,475	Proteinuria as protein excretion >0.5 g/24 h	Cumulative incidence of proteinuria 45% after 40 years Two incidence peaks of proteinuria: at 16 and 32 years of diabetes duration
Krolewski et al., 1985 (10)	Single-center (Joslin Clinic, the United States)	292	Proteinuria as protein excretion ≥1.0 g/24 h or urinary protein concentration ≥30 mg/dl	Cumulative incidence of proteinuria 35% after 40 years One incidence peak of proteinuria at 10-14 years of diabetes duration
Borch-Johnsen et al., 1985 (11)	Single-center (Steno Memorial Hospital, Denmark)	1,001	Proteinuria as protein excretion >0.5 g/24 h	One incidence peak of proteinuria at 13-18 years of diabetes duration
Viberti et al., 1982 (12)	Single-center (Guy’s Diabetic Clinic, the United Kingdom)	63 (55 with initial normal AER; 8 with initial moderate albuminuria)	Moderate albuminuria as AER >30 µg/min Proteinuria as dipstick-positive protein excretion (corresponding to ≥0.5 g/24 h)	7/8 individuals with initial moderate albuminuria developed proteinuria over 14 years of follow-up
Parving et al., 1982 (13)	Single-center (Steno Memorial Hospital, Denmark)	25 (17 with initial normal AER; 8 with initial moderate albuminuria)	Moderate albuminuria as >40 µg/min Proteinuria as protein excretion >0.5/24 h	5/8 individuals with initial moderate albuminuria developed proteinuria over 6 years of follow-up
Mogensen et al., 1984 (14)	Single-center (Aarhus, Denmark)	43 (29 with initial normal AER; 14 with initial moderate albuminuria)	Moderate albuminuria as ≥15 µg/min Proteinuria as protein excretion >0.5 g/24 h or AER >150 µg/min	12/14 individuals with initial moderate albuminuria developed proteinuria over 10 years of follow-up
Moderate albuminuria				
Hovind et al., 2004 (15)	Single-center (Steno Diabetes Center, Denmark)	222	Moderate albuminuria as AER 30-300 mg/24 h	Cumulative incidence of moderate albuminuria after a mean follow-up of 18 years 33.6%
Amin et al., 2008 (16)	Multi-center regional (Oxford regional prospective study, the United Kingdom)	527	Moderate albuminuria as albumin-creatinine ratio 3.5-35 mg/mmol in men and 4.0-47 mg/mmol in women	10-year cumulative incidence of moderate albuminuria 25.7% 19-year cumulative incidence of moderate albuminuria 50.7%
Costacou et al., 2018 (17)	Single-center (Pittsburgh Epidemiology of Diabetes Complications study; Children’s Hospital of Pittsburgh, the United States)	658	Moderate albuminuria as AER 20-200 µg/min or 30-300 mg/24 h	No change over time in the cumulative incidence of moderate albuminuria: Cumulative incidence at 30, 40, and 50 years for the 1950-1964 diagnosis cohort: 65.2%, 79.0%, and 88.0%, respectively Cumulative incidence at 20, 30, and 40 years in the 1965-1980 diagnosis cohort: 54.7%, 70.0%, and 81.7%, respectively
Goñi et al., 2016 (18)	Single-center (Navarra Hospital Complex, Spain)	716	Moderate albuminuria as AER 20-200 mg/g	5-year cumulative incidence of moderate albuminuria 2.5% 10-year cumulative incidence of moderate albuminuria 6.1% 15-year cumulative incidence of moderate albuminuria 10.6%
Esmatjes et al., 2002 (19)	Multicenter national (Estudio Diamante, Spain)	1,225	Moderate albuminuria as AER 20-200 µg/min	Moderate albuminuria annual incidence rate 2.7%
Stone et al., 2006 (20)	Single-center (The Children’s Hospital at Westmead, Australia)	972	Moderate albuminuria as AER 20-200 µg/min	Moderate albuminuria incidence rate 4.6 per 1,000 patient-years
Jansson Sigfrids et al., 2022 (21)	Nationwide, population-based (Finland)	961	Moderate albuminuria as AER ≥30 mg/24 h and <300 mg/24 h, or ≥20 mg/24 h and <200 mg/24 h, or albumin-creatinine ratio ≥3 mg/mmol and <30 mg/mmol	25-year cumulative incidence of moderate albuminuria (diagnosis cohort 1980-1989) 29.8% 25-year cumulative incidence of moderate albuminuria (diagnosis cohort 1990-1999) 30.7% No reduction in the cumulative incidence between 1980-89 and 1990-1999 diagnosis cohorts Mean incidence rate between 10 and 24 years of diabetes duration 19.2 per 1,000 person-years
Severe albuminuria				
Krolewski et al., 1985 (10)	Single-center (Joslin Clinic, the United States)	292	Proteinuria as protein excretion ≥1.0 g/24 h or urinary protein concentration ≥30 mg/dl	Declining temporal trend (risk of proteinuria halved between 1939 and 1949 & 1959 diagnosis cohorts) Peak incidence rate 25.1 per 1,000 person-years (for 10-14 years of diabetes duration)
Kofoed-Enevoldsen et al., 1987 (22)	Single-center (Steno Memorial Hospital, Denmark)	2,454	Proteinuria as protein excretion >0.5 g/24 h	Declining incidence of proteinuria over time: 25-year cumulative incidence 40.6% and 26.9% for the 1933-1942 and 1953-1962 diagnosis cohorts, respectively
Hovind et al., 2003 (23)	Single-center (Steno Diabetes Center, Denmark)	600	Severe albuminuria as AER >300 mg/24 h	Declining incidence of severe albuminuria over time: 20-year cumulative incidence 31.1%, 28.4%, 18.9%, and 13.7% for the 1965-69, 1970-74, 1975-79, and 1979-1981 diagnosis cohorts, respectively
Nordwall et al., 2004 (24)	Single-center (Linköping University Hospital, Sweden)	269	Severe albuminuria as 1+ test result with semi-quantitative dipstick method (corresponding to urinary albumin concentration ≥300 mg/l) or, in later decades, as AER ≥200 µg/min or urinary albumin concentration ≥300 mg/l (quantitative method)	Declining incidence of severe albuminuria over time: 20-year cumulative incidence 30.3%, 8.2%, and 9.2% for the 1961-65, 1966-70, and 1971-75 diagnosis cohorts, respectively
Tryggvason et al., 2005 (25)	Nationwide, population-based (Iceland)	343	Proteinuria as 1+ test result with semi-quantitative dipstick method (corresponding to urinary protein concentration ≥0.3 g/l)	No change over time in the cumulative incidence of proteinuria: 20-year cumulative incidence 24.0%, 26.1%, 24.3%, 13.4%, and 25.9% for the -1960, 1961-1965, 1966-1970, 1971-1975, and 1976-1980 diagnosis cohorts, respectively Peak incidence 23.8 per 1,000 person-years (at 15–19 years of diabetes duration)
Costacou et al., 2018 (17)	Single-center (Pittsburgh Epidemiology of Diabetes Complications study; Children’s Hospital of Pittsburgh, the United States)	658	Severe albuminuria as AER >200 µg/min or >300 mg/24 h	No change over time in the cumulative incidence of severe albuminuria: Cumulative incidence at 30, 40, and 50 years for the 1950-1964 diagnosis cohort: 43.3%, 57.1%, and 71.8%, respectively Cumulative incidence at 20, 30, and 40 years for the 1965-1980 diagnosis cohort: 27.1%, 39.9%, and 57.3%, respectively
Jansson Sigfrids et al., 2022 (21)	Nationwide, population-based (Finland)	1,430	Severe albuminuria as AER ≥300 mg/24 h or ≥200 µg/min or albumin-creatinine ratio ≥30 mg/mmol	25-year cumulative incidence of severe albuminuria (diagnosis cohort 1970-1979) 26.8% 25-year cumulative incidence of severe albuminuria (diagnosis cohort 1980-1989) 12.0% 25-year cumulative incidence of severe albuminuria (diagnosis cohort 1990-1999) 10.8% Significant reduction in the cumulative incidence between 1970-79 and 1980-89 diagnosis cohorts, but not between 1980-89 and 1990-99 Shift in duration-specific incidence rate over time: incidence peak between 15 and 19 years in the earliest diagnosis cohort (25.8 cases per 1,000 person-years) but not in the later ones.
Progression of albuminuria				
de Boer et al., 2011 (26)	Multi-center clinical trial (DCCT/EDIC, the United States)	325	Moderate albuminuria as ≥30 mg/24 h and <300 mg/24 h Severe albuminuria as ≥300 mg/24 h	10-year cumulative progression rate from moderate to severe albuminuria 28%
de Boer et al., 2014 (27)	Multi-center clinical trial (DCCT/EDIC, the United States)	159	Severe albuminuria as AER ≥300 mg/24 h	10-year cumulative progression rate from severe albuminuria to kidney failure 16%
Jansson Sigfrids et al., 2022 (21)	Nationwide, population-based (Finland)	273 with initial moderate albuminuria, 273 with initial severe albuminuria	Moderate albuminuria as AER ≥30 mg/24 h and <300 mg/24 h, or ≥20 mg/24 h and <200 mg/24 h, or albumin-creatinine ratio ≥3 mg/mmol and <30 mg/mmol Severe albuminuria as AER ≥300 mg/24 h or ≥200 µg/min or albumin-creatinine ratio ≥30 mg/mmol	10-year cumulative progression rate from moderate to severe albuminuria 44.3% 10-year cumulative progression rate from severe albuminuria to kidney failure 23.5% and 28.0% in the 1970-79 and 1980-99 diagnosis cohorts, respectively (death as competing risk)
Regression of albuminuria				
Forsblom et al., 1992 (28)	Single-center (Outpatient clinic of Helsinki University Hospital, Finland)	42 with initial moderate or severe albuminuria	Moderate albuminuria as AER 20-200 µg/min or 30-300 mg/24 h Severe albuminuria as AER >200 µg/min or >300 mg/24 h	20 had initial moderate albuminuria; 13 had sustained moderate albuminuria and 5 progressed to severe albuminuria during the 10-year follow-up 22 had initial severe albuminuria; 3 had regressed to moderate albuminuria, 12 had sustained severe albuminuria, and 7 had progressed to kidney failure during the 10-year follow-up
Bojestig et al., 1996 (29)	Single-center (Linköping University Hospital, Sweden)	27 with initial moderate albuminuria	Moderate albuminuria as AER 20-200 µg/min or 30-300 mg/24 h	Regression rate from moderate albuminuria to normal AER 58% (10-year follow-up)
Perkins et al., 2003 (30)	Single-center (Joslin Clinic, the United States)	386	Moderate albuminuria as AER 30-299 µg/min or 43-430 mg/24 h	Regression rate from moderate albuminuria to normal AER 58% (6-year follow-up)
Giorgino et al., 2004 (31)	Multi-center, multinational (EURODIAB Prospective Complications Study)	352	Moderate albuminuria as AER 20-200 µg/min	Regression rate from moderate albuminuria to normal AER 50.6% (7-year follow-up)
Amin et al., 2008 (16)	Multi-center regional (Oxford regional prospective study, the United Kingdom)	135	Moderate albuminuria as albumin-creatinine ratio 3.5-35 mg/mmol in men and 4.0-47 mg/mmol in women	Regression rate from moderate albuminuria to normal AER 51.9% (5-year follow-up) Relapse rate to moderate albuminuria 24%
Steinke et al., 2005 (32)	Multi-center, multinational (Natural history study)	22 with initial moderate albuminuria	Moderate albuminuria as AER 20-200 µg/min	Regression rate from moderate albuminuria to normal AER 64%
Galler et al., 2012 (33)	Multi-center, multinational (German and Austrian diabetes survey)	59 with initial moderate albuminuria	Moderate albuminuria as AER ≥20 µg/min and <200 µg/min or albumin-creatinine ratio ≥2.5 mg/mmol and <35 mg/mmol	Regression rate from moderate albuminuria to normal AER 71.2% (5-year follow-up)
Hovind et al., 2003 (23)	Single-center (Steno Diabetes Center, Denmark)	79 with initial moderate albuminuria	Moderate albuminuria as AER 30-300 mg/24 h	Regression rate from moderate albuminuria to normal AER 35.4% (7.5-year follow-up) Relapse rate to moderate albuminuria after regression 54%
Rossing et al., 2005 (34)	Single-center (Steno Diabetes Center, Denmark)	334 with initial moderate albuminuria	Moderate albuminuria as AER 31-299 mg/24 h	Regression rate from moderate albuminuria to normal AER 16% (10-year follow-up)
Vaidya et al., 2011 (35)	Single-center (Second Joslin Kidney Study; Joslin Clinic, the United States)	296 with initial moderate albuminuria	Moderate albuminuria as AER 30-300 µg/min	Regression rate from moderate albuminuria to normal AER 24% (2-year follow-up)
Bjornstad et al., 2015 (36)	Multi-center, national (CACTI study; the United States)	81	Albuminuria as AER ≥20 µg/min	Regression rate from albuminuria to normal AER 38% (6-year follow-up)
Jansson et al., 2018 (37)	Multi-center, national (FinnDiane Study, Finland)	438 with initial moderate albuminuria, 475 with initial severe albuminuria	Moderate albuminuria as AER 30-300 mg/24 h or 20-200 µg/min, or albumin-creatinine ratio 2.5-25 mg/mmol for men and 3.5-35 mg/mmol for women	Regression rate 23% from both moderate and severe albuminuria Regression was associated with reduced cardiovascular disease and premature mortality risk
de Boer et al., 2016 (38)	Multi-center clinical trial (DCCT/EDIC, the United States)	423 with initial moderate albuminuria	Moderate albuminuria as AER ≥30 mg/24 h and <300 mg/24 h	Regression rate from moderate albuminuria to normal AER 40% Relapse rate to moderate albuminuria after regression 25% Regression was not associated with a reduced cardiovascular disease risk
Goñi et al., 2016 (18)	Single-center (Navarra Hospital Complex, Spain)	53 with initial moderate albuminuria	Moderate albuminuria as AER 20-200 mg/g	Regression rate from moderate albuminuria to normal AER 36% Relapse rate to moderate albuminuria after regression 100% over 5 years
Kidney failure				
Möllsten et al., 2010 (39)	Nationwide, population-based (Sweden)	11,681	*NA*	No change over time in the cumulative incidence of kidney failure (1977–1995) 30-year cumulative incidence 3.3%
Otani et al., 2016 (40)	Single-center (Diabetes center of Tokyo Women’s Medical University, Japan)	1,014	*NA*	Declining incidence of kidney failure over time (1961-1984 vs. 1985-1999) 25-year cumulative incidence 3.0% (diagnosis cohort 1985-1999)
Gagnum et al., 2018 (41)	Nationwide, population-based (Norway)	7,871	*NA*	No change over time in the cumulative incidence of kidney failure (1973-1982 vs. 1989-2012) 30-year cumulative incidence 2.9% (diagnosis cohorts 1973-1982 and 1989-2012)
Helve et al., 2018 (42)	Nationwide, population-based (Finland)	29,906	*NA*	Declining incidence of kidney failure over time (1965–2011) 30-year cumulative incidence 4.4% (diagnosis after 1980)
Costacou et al., 2018 (17)	Single-center (Pittsburgh Epidemiology of Diabetes Complications study; Children’s Hospital of Pittsburgh, the United States)	932	*NA*	Declining incidence of kidney failure over time: Cumulative incidence at 20, 30, 40, and 50 years for the 1950-1964 diagnosis cohort: 14.5%, 34.6%, 48.5%, and 61.3%, respectively Cumulative incidence at 20, 30, and 40 years for the 1965-1980 diagnosis cohort: 5.5%, 14.5%, and 26.5%, respectively

AER, albumin excretion rate. NA, Not applicable.

## Proteinuria, albuminuria, and categorization of diabetic kidney disease

Proteinuria, as assessed by dipstick tests, was initially used to reveal the presence of kidney disease in individuals with diabetes. However, the dipstick tests were limited to finding substantial quantities of proteins excreted in the urine, typically exceeding several grams per day – thus, only catching far-advanced disease manifestations. The first immunoassay method for measuring urinary albumin was launched in 1963 (43), but it was not until the 1980s when microalbuminuria – as it was coined by Viberti et al. (nowadays moderate albuminuria) – was distinguished as the first detectable clinical expression of diabetic kidney disease. This finding marked a significant milestone in the early diagnostics of diabetic kidney disease.

Originally, the gold standard for screening albuminuria in clinical practice was albumin measurements from 24-hour urine collections. However, the arduous nature of this process, leading to potential inaccuracies in timekeeping and urinary volume measurement, led to a shift in the preferred method. Timed overnight urine collections were subsequently favored due to their ease of implementation over the 24-hour specimens. However, they were still considered cumbersome. Therefore, when albuminuria testing became a standard component of care for individuals with type 2 diabetes, the American Diabetes Association recommended albumin-creatinine ratios derived from spot urine collections as the new gold standard for albuminuria screening in diabetes. Albumin-creatinine ratios – or some other validated measurement of albumin excretion – should be monitored at least annually after a diabetes duration of five years has been reached in individuals with type 1 diabetes. Albuminuria is graded as moderately increased when the albumin-creatinine ratio is 3–30 mg/mmol or 30–300 mg/g, or the albumin excretion rate (AER) is 30–300 mg/24 h or 20–200 µg/min. The more advanced stage, severe albuminuria, is defined as an albumin-creatinine ratio of >30 mg/mmol or >300 mg/g, or an AER of >300 mg/24 h or >200 µg/min.

Beyond albuminuria, diabetic kidney disease is characterized by elevated blood pressure, a heightened risk of cardiovascular disease, and reduced kidney function. Yet, also glomerular hyperfiltration, or elevated glomerular filtration rate (GFR) into supraphysiological levels, is noted in as many as two out of three individuals with type 1 diabetes – particularly during initial stages of the disease and among those with inadequate glycemic control (44). Hyperfiltration is hypothesized to contribute to the development of more advanced kidney disease as well as adverse cardiovascular outcomes (45, 46), however, the available data are inconsistent (47, 48).

In diabetic kidney disease, a rise in blood pressure typically occurs early in the clinical course, around the same stage when albumin first appears. As the disease progresses, albuminuria increases to the stage of severe albuminuria (previously referred to as macroalbuminuria), and kidney function starts to decline. Reduced kidney function can also occur without albuminuria; but unlike in type 2 diabetes, the prevalence of this disease type has been rather low in most study settings involving individuals with type 1 diabetes (49–51). Thus, this review will focus on the “traditional” course of diabetic kidney disease in type 1 diabetes with elevated albumin excretion at its core.

## The early epidemiology of albuminuria in type 1 diabetes

The first detailed descriptions of the epidemiology of albuminuria (initially as proteinuria) in type 1 diabetes can be traced to the 1980s, reporting data on individuals with diabetes diagnosed during the first half of the 20th century. Two of the early studies, conducted at the Steno Memorial Hospital in Denmark (9) and the Joslin Clinic in the United States (10), uncovered a cumulative incidence of persistent proteinuria ranging from 25 to 35% after 25 years of diabetes and 35 to 45% after 40 years. The Danish study by Andersen et al. identified two peaks in the incidence rate of proteinuria; the first and larger peak emerged after 16 years of diabetes, while the second one manifested at around 32 years (9). Krolewski et al. at the Joslin Clinic found that the incidence reached its highest point 10 to 14 years after the onset of diabetes, whereas they could not replicate the presence of a second peak (10). Likewise, another study conducted later at the Steno Memorial Hospital using slightly different inclusion criteria than the one mentioned above only observed one incidence peak of proteinuria, between 13 and 18 years of diabetes duration (11). The characteristic incidence rate pattern was hypothesized to mirror a genetic predisposition to diabetic kidney disease; if proteinuria developed only due to hyperglycemia, the incidence would continue to rise uniformly until most individuals were affected.

The first studies on moderate albuminuria (previously called microalbuminuria) were also published in the early 1980s. Three seminal studies by Viberti (12), Parving (13), and Mogensen (14) et al. provided a gloomy outlook for individuals with microalbuminuria: During 6–14 years, 63–88% of the participants developed persistent proteinuria. Microalbuminuria was also found to pose a marked upsurge in the mortality risk. Although these studies were small-scale (altogether 30 participants), they led to the widespread assumption that progression is virtually unavoidable if microalbuminuria occurs, which engendered a new era in diabetes research and clinical practice.

## Recent discoveries in the incidence of moderate albuminuria

As methods to detect microalbuminuria were not widely accessible until the 1980s, data portraying the epidemiology of the earliest stage of albuminuria are naturally only available from the past 40 years. In addition, most studies on the topic have had crucial shortcomings, and therefore, the true incidence rates and cumulative incidences of moderate albuminuria were long unexplored. The study shortcomings have been as follows: First, most studies have not followed the participants from their diabetes diagnoses onwards but instead recruited individuals with normal albumin excretion rates (AERs) after several years (up to 20 or more) of diabetes duration, which provokes a risk of selection bias. Second, the follow-up times have mostly been short. As it is known that new cases of moderate albuminuria appear even 30 to 40 years after the onset of diabetes, the limited follow-up times can undoubtedly lead to many diagnoses being overlooked. Third, studies have predominantly been small-scale with single-center settings and, thus, not generalizable to larger populations of individuals with type 1 diabetes. Lastly, only a few studies have assessed temporal patterns, that is, fluctuations in the incidence rate over time.

Four studies have analyzed the cumulative incidences of moderate albuminuria in individuals with newly-onset diabetes at the study initiation, yet with single-center study designs or, as in one case, a multi-center regional study design. Three of these studies measured the AER at least annually, whereas one did so biennially. In the first study, Hovind et al. followed 222 individuals, at the Steno Diabetes Center in Denmark, from their diabetes diagnoses in 1979-1984 for a median of 18 years (range 1.0 to 21.5 years) until December 2000 (15). The 18-year cumulative incidence of moderate albuminuria was 33.6% (95% CI 27.2–40.0), with the first cases already appearing before a diabetes duration of five years had been reached. Amin et al. studied 527 individuals with childhood-onset diabetes from 1986-1997 until a mean diabetes duration of 9.8 years (9% had over 15 years of follow-up) as part of the Oxford regional prospective study (16). The cumulative incidence of moderate albuminuria after ten years was 25.7% (95% CI 21.3–30.1) and 50.7% (95% CI 40.5–60.9) after 19 years in the cohort; that is, higher than in the study by Hovind et al. after an almost similar follow-up period. Yet, it is in line with the 20-year cumulative incidence of 54.7% that was reported from the Pittsburgh Epidemiology of Diabetes Complications (EDC) study (17). In the Pittsburgh EDC study, the study participants had been diagnosed with type 1 diabetes between 1950 and 1980 (but the 20-year cumulative incidence was only calculated from those with diabetes diagnosed after 1965) and were regularly examined for up to 50 years. After 30 years, the cumulative incidence of moderate albuminuria was 65.2% in the 1950-64 and 70.0% in the 1965–80 cohort; and it was 79.0% and 81.7%, respectively, after 40 years. The cumulative incidence was as high as 88.0% after 50 years in those diagnosed with diabetes in 1964 or prior to that. Notably, there was no difference in the incidences between the two cumulative incidence cohorts. Lastly, cumulative incidences were calculated in a Spanish single-center cohort comprising over 700 individuals with diabetes since 1991–2008 (18). The 10-year cumulative incidence of 6.1% (95% CI 4.0–9.1) was considerably lower than in the Oxford regional prospective study, despite many similarities in study demographics. The 15-year cumulative incidence was also only 10.6% (7.4–13.6), but due to a limited follow-up time, incidence rates after longer diabetes durations were not reported in that study.

Aside from cumulative incidences, some studies have also reported annual incidence rates of moderate albuminuria in type 1 diabetes. The incidence rate per annum was 2.7% (95% CI 2.2–3.2) in the Spanish multicenter Estudio Diamante Study (19). At the study baseline in 1995, the participants’ mean diabetes duration was 14.1 years, and they were followed for 4.3 years longitudinally. Yet in another study by Stone et al., the incidence rate was only 4.6 per 1,000 patient-years (20). As the incidence rate of albuminuria is known to be uneven throughout the diabetes life-span, the discrepancy between the analyses is presumably – at least partly – explained by differences in study characteristics: at the baseline (1989–2004) of the study by Stone et al., the median diabetes duration was only 6.5 years (interquartile range 4.1–9.3). In other words, the reported incidence rate was for many participants for an earlier phase of their diabetes course. The follow-up time was also slightly longer than in the Estudio Diamante Study (6.2 years).

Thus, to explore updated incidence rate patterns, and to calculate diabetes duration-specific incidence rates, as well as to overcome the other limitations listed above, we recently conducted a nationwide population-based study aiming to comprehensively investigate the epidemiology of diabetic kidney disease in Finland – the country with the highest incidence of type 1 diabetes in the world (21). The study observed individuals with type 1 diabetes from their diabetes diagnoses onwards for a median of 21.8 years (but even up to 35 years). Regarding moderate albuminuria, two diagnosis cohorts were formed: one with diabetes diagnosed in 1980–89 and the other in 1990–99. However, the incidence rate pattern was similar in the two diagnosis cohorts, and therefore, incidence rates were calculated for all participants jointly.

In the study, we found that the incidence rate rose until 10 years of diabetes duration; it was then rather stable until 25 years of duration, whereafter it started to decrease. Between 5 and 9 years after the onset of diabetes, the incidence rate was 13.5 (95% CI 10.4–17.3) cases per 1,000 person-years (*previously unpublished data*). The average incidence rate between 10 and 24 years was 19.2 (95% CI 16.5–22.2) per 1,000 person-years.

**Figure 1:**
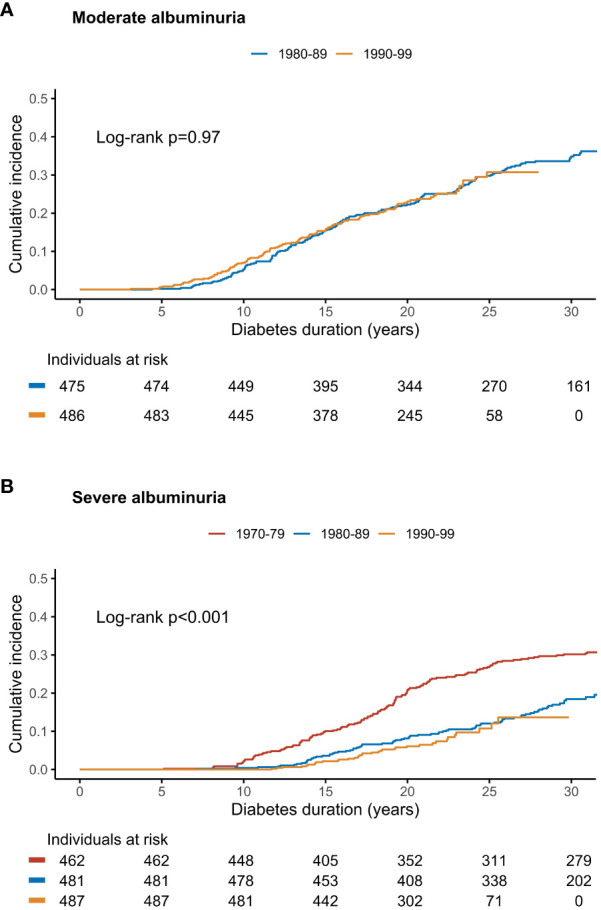
The cumulative incidence of **(A)** moderate and **(B)** severe albuminuria stratified by cohort of diabetes diagnosis in a Finnish nationwide, population-based study. Log-rank p for between-group difference. Adapted from (21) with permission from Elsevier.

We also calculated cumulative incidences of moderate albuminuria. Notably, there was no change in the cumulative incidence over time between the two diagnosis cohorts (hazard ratio 0.99 [95% CI 0.78–1.28] with the 1980s cohort as a reference), as the incidence curves tracked each other almost identically (**Figure 1A**). The cumulative incidences of moderate albuminuria at 15 years were 15.5% (95% CI 12.2-18.7) and 15.6% (12.2–18.8) in the 1980s and 1990s cohorts, respectively. At 25 years, they were 29.8% (25.4-33.9) and 30.7% (24.8–36.2). The follow-up extended until 35 years of diabetes in the earlier 1980s cohort: at this point, the cumulative incidence had risen to 38.6% (32.7–43.9).

## Temporal trends of severe albuminuria

Over the past decades, a few studies have shed light on the temporal trends of severe albuminuria (previously macroalbuminuria) in type 1 diabetes. The first paper to reveal a declining incidence of proteinuria was, in fact, already published in the 1980s. In this pioneering work by Krolewski et al. (10), individuals diagnosed with type 1 diabetes in the late 1930s exhibited twice the risk of proteinuria compared to their more recently (late 1940s and -1950s) diagnosed counterparts. Building upon these observations, Kofoed-Enevoldsen et al. from the Steno Memorial Center conducted a comprehensive evaluation of individuals diagnosed with type 1 diabetes between 1933 and 1972, reporting cumulative incidence rates after a long follow-up (22). For those diagnosed between 1933 and 1942, the 25-year incidence of proteinuria stood at 40.6%, but this figure significantly decreased to 26.9% for those diagnosed between 1953 and 1962.

The notion of an improving prognosis for severe albuminuria in type 1 diabetes was further reinforced by another Danish study, as Hovind et al. revealed marked differences in the 20-year cumulative incidence rates over time (23). Specifically, incidence rates were found to be 31.1%, 28.4%, 18.9%, and 13.7% for diagnosis cohorts 1965-69, 1970-74, 1975-79, and 1979-1984, respectively, with the trend significantly decreasing. Results from the neighboring country, Sweden, paralleled these encouraging findings: The 20-year cumulative incidences showcased a similar declining pattern at 30.3%, 8.2%, and 9.2% for diagnosis cohorts 1961-65, 1966-70, and 1971-75, respectively (24).

However, the picture is not entirely uniform. An Icelandic nationwide study comprising virtually all individuals with type 1 diabetes over four decades (up to 1992) did not discover any changes in the incidence over time (25). The cumulative incidence at 20 years hovered around 25% for all diagnosis cohorts from before 1961 to 1980, except for the 1971-1975 cohort, in which it was as low as 13.4%. However, this difference was not statistically significant and is likely explained by the loss of follow-up for a substantial part of the study participants in this particular 5-year diagnosis cohort. Similarly, the Pittsburgh EDC study did not reveal evidence of a decline in the cumulative risk of severe albuminuria between the diagnosis cohorts of 1950-64 and 1965-80 (17). At 30 years, the cumulative incidence of severe albuminuria stood at 43.3% and 39.9% in the 1950-64 and 1965-80 cohorts (p for between-group difference =0.45), respectively, while at 40 years, it had reached 57.1% and 57.3% (p=0.96). At 50 years, the incidence had exceeded 70% in the earlier diagnosis cohort.

The most recent data on temporal trends of severe albuminuria in type 1 diabetes stem from our Finnish population-based study comparing three diagnosis cohorts: 1970–79, 1980–89, and 1990–99 (21). A remarkable halving of the cumulative incidence of severe albuminuria was observed between the 1970–79 and 1980–89 cohorts, with a hazard ratio of 0.55 (95% CI 0.42–0.72), p<0.0001, compared to the 1970s cohort. At 25 years, the cumulative incidence was 26.8% (95% CI 22.6–30.8) in the earlier cohort and 12.0% (9.0–15.0) in the latter. Unfortunately, there was no further reduction after this: In individuals diagnosed during the 1990s, the 25-year cumulative incidence was 10.8% (6.7-14.6), and the hazard ratio 0.83 (0.54–1.26, p=0.38) with the 1980s cohort as reference (**Figure 1B**).

To the best of our knowledge, our study is thus far the only one to explore temporal changes in the diabetes duration-specific incidence rate patterns of severe albuminuria after the early 1980s studies (21). Interestingly, we did not only uncover a drop in the cumulative incidence after the 1970s but also a clear shift in the incidence rate pattern after the 1970s – but, again, the picture was no different after the 1980s. In the earliest diagnosis cohort, we found a clear incidence peak between 15 and 19 years of diabetes duration; whereafter it decreased and remained at a lower level for the rest of the follow-up. The peak incidence rate (at 15–19 years of follow-up) was 25.8 cases (95% CI 19.1–34.1) per 1,000 person-years. It is noteworthy that in the early study by Krolewski et al. (10), examining individuals diagnosed with type 1 diabetes since the 1930s–1950s, the highest incidence rate per 5-year interval of diabetes duration was 25.1 per 1,000 person-years (for 10-14 years of diabetes duration) – that is, very similar to the one published from our, more recent cohort. The highest duration-specific incidence was also 23.8 per 1,000 person-years (at 15–19 years of diabetes duration) in the nationwide Icelandic study referred to above (25). However, in our study’s combined 1980s and 1990s cohort, no peak in the duration-specific incidence rate could be seen anymore, as the initial increase in incidence plateaued after 14 years of diabetes. Subsequently, the average incidence rate between 15 and 34 years after the diabetes onset remained stable at 10.2 cases (95% CI 8.2–12.6) per 1,000 person-years, showing no significant decrease over time. Yet, it is noteworthy that at 25 to 29 years of diabetes duration, the incidence rate ratio was 2.27 for the 1980–99 cohort with the 1970–79 as reference. This raises the question of whether we are preventing – or solely postponing the onset – of severe albuminuria in modern times.

## Progression of moderate and severe albuminuria is common and of great concern

Progression rates of albuminuria have been extensively studied, for instance, in the groundbreaking DCCT/EDIC study. The rationale of the DCCT (Diabetes Control and Complications Trial) was to determine whether maintaining strict blood glucose control could reduce the risk of diabetes-related complications, thereby providing crucial insights into treatment strategies for type 1 diabetes that remain to this day. The EDIC (Epidemiology of Diabetes Interventions and Complications) Study continued monitoring the long-term effects of intensive versus conventional diabetes management after the conclusion of the DCCT trial (52). During the DCCT/EDIC, 325 participants developed incident moderate albuminuria, and the median follow-up time after a diagnosis of moderate albuminuria was 13 years. The ten-year cumulative incidence of progression to severe albuminuria was 28% – that is, considerably lower than in the first reports published in the early 1980s. The prevalence of severe albuminuria increased during the first four years following the initiation of moderate albuminuria, whereafter the progression rate stabilized to a flatter incline. Progression was less common in the intensive than in the conventional treatment arm (26).

The DCCT/EDIC further evaluated long-term kidney outcomes among those 159 individuals, who developed severe albuminuria, following them for a median of 9 years. Notably, although regression from this advanced stage of kidney disease has also been pinpointed during recent years, it has traditionally been viewed as a step on a somewhat irrevocable path toward GFR loss. In the DCCT/EDIC, the ten-year cumulative incidences of sustained eGFR <60, <45, and <30 mL/min per 1.73 m2 after the severe albuminuria diagnosis were 32%, 26%, and 22%, respectively. The ten-year cumulative incidence of kidney failure (i.e., need for kidney replacement therapy) was 16%. Again, unfavorable kidney disease outcomes were more common in the conventional treatment arm (27).

Interestingly, the progression rates presented above differ vastly from those uncovered in our recent, population-based Finnish study (21). In our combined 1980–89 and 1990–99 diagnosis cohorts, the cumulative progression to severe albuminuria was 32.2% (95% CI 26.1–37.9) five years after the initial onset of moderate albuminuria – thus, already at this stage higher than the 10-year cumulative progression rate of 28% in the DCCT/EDIC. The 10-year cumulative progression rate was 44.3% (37.3%–50.6%) in our cohort (*previously unpublished data*). At 15 years, it had even exceeded 50% (54.3% [46.0–61.3]). As in the DCCT/EDIC study, the steepest incline in the cumulative progression appeared during the first years after the initial debut of moderate albuminuria.

Progression from severe albuminuria to kidney failure was evaluated with death as a competing risk. The two diagnosis cohorts 1970–79 and 1980–99 were studied separately; however, no differences between these were found (Gray’s test p=0.37). The 10-year progression rate was 23.5% in the 1970-1979 cohort and 28.0% in the 1980-99 cohort (*previously unpublished data*); hence, also higher than in the DCCT/EDIC. Fifteen years after the onset of severe albuminuria, the progression rates were 35.2% (27.4–43.0) and 35.6% (24.3–47.0), respectively. Reasons for the discrepancy between the DCCT/EDIC study and ours remain unknown; however, it is plausible that they stem from differences in study design and treatment approaches (intervention-based investigation with strict selection criteria vs. real-world study). Nevertheless, despite the rates being lower than discovered in the early reports from the 1980s, progression of albuminuria still occurs in a significant share of individuals with type 1 diabetes – and, importantly yet alarmingly, this proportion shows no sign of reduction since the 1970s.

Why is the progression to more advanced stages of diabetic kidney disease a matter of concern? The answer is that the risk of encountering adverse comorbidities grows progressively as kidney disease advances. For coronary artery disease, the risk surges to 1.52-fold in cases of moderate albuminuria and further to 2.3-fold in those with severe albuminuria, as indicated by adjusted estimates, when compared against normal AER (53). In comparison to the general population without diabetes, the incidence rate rockets to 6.3-fold and 13.1-fold, respectively. The realm of stroke paints a similar picture. The hazard ratio against normal AER span from 2.5-fold to 3.2-fold in moderate albuminuria, and rise to 2.7 to 4.9-fold in severe albuminuria (53, 54). The standardized incidence ratio (that is, compared to the non-diabetic population) is 5.8-fold and 10.1-fold, respectively (53).

As cardiovascular causes of death prevail in individuals with diabetes [after the first decade of diabetes, when the mortality is driven by acute diabetes complications (6, 55)], it is no surprise that this pattern extends to the mortality risk. In cases of moderate albuminuria, all-cause mortality stands at 2.8 times that of normal AER, whereas in severe albuminuria, it reaches a magnitude of 5.9. The standardized mortality ratio is up to 6.4-fold and 12.5-fold, respectively (56, 57). It is further no surprise that these risk estimates culminate in kidney failure – the most advanced stage of diabetic kidney disease. For coronary artery disease, the standardized incidence ratio surges to a remarkable 26.6, while for stroke, it stands at 20.7 (53). The standardized mortality ratio also approaches a 20-fold magnitude – in some populations nearly 30-fold (56, 57).

## Regression of albuminuria is prevalent in contemporary times

Only a few years after Viberti (12), Parving (13), and Mogensen’s (14) landmark discoveries suggesting relentless progression after the first signs of albuminuria, studies detecting unchanged or even reverted albuminuria over time began appearing in the academic literature. In 1992, pioneering work by Forsblom et al. questioned the high kidney disease progression rates that had been published before, stating that moderate albuminuria is not a good predictor of future overt kidney disease after all (28). In the study by Forsblom et al., moderate albuminuria progressed only in 28% of the individuals, and persisted in the others. Among the first ones to demonstrate albuminuria regression were Bojestig et al., who found a regression rate as high as 58% (15 of 26 participants) over a 10-year follow-up in a single-center cohort of individuals with the onset of diabetes between 1961 and 1980 (29). A groundbreaking study from the Joslin Center replicated these findings in a larger cohort in 2003, also presenting a regression rate of 58% from moderate albuminuria in type 1 diabetes (study participants N=386) (30). The study defined regression as a 50-percent reduction in AER between two 2-year observation periods; however, strikingly, the regression rate was almost identical (59%) when defining regression as a change from moderate albuminuria to normal-range AER, in line with Bojestig et al.

Since the studies by Bojestig and Perkins with colleagues, also others have evaluated the natural history of albuminuria in type 1 diabetes. The studies have varied substantially regarding study designs, the number of participants, and follow-up protocols – and, importantly, the definition of albuminuria regression. It is noteworthy that no universally accepted definition exists, and thus, both categorical (change from moderate albuminuria to normal AER) and continuous (such as a 30 or 50% reduction in AER) ones have appeared. Regardless of these discrepancies, the conclusion of regression being the most frequent outcome of moderate albuminuria has been drawn repeatedly. The EURODIAB Prospective Complications Study, a collaboration between 31 centers in 16 European countries, found that 50.6% of the initial 352 individuals with type 1 diabetes and moderate albuminuria at baseline regressed to normal AER over a 7-year follow-up (31). Similarly, 4.9 years after the onset of albuminuria, 51.9% of the 135 evaluated children had undergone albuminuria regression in the Oxford regional prospective study (16). The Natural History study, which aimed to assess different patterns of albuminuria, found a regression rate of 64% (14 of 22 individuals) at the end of their 5-year follow-up phase (32). In the German and Austrian diabetes survey, the regression rate after five years had even exceeded 70% (initially N=59 with moderate albuminuria) (33).

Nevertheless, lower regression rates – typically in the scope of 15 to 35% – have also appeared in the literature (15, 34–37). Notably, most reports presented above arise from rather small study cohorts, which may partly explain the dissimilarities.

What is also noteworthy is the seemingly high relapse rate from regression back to the more advanced stage of albuminuria. In other words, albuminuria is labile to its nature – especially early during the course of diabetes and diabetic kidney disease. For instance, in the study by Hovind et al. reviewed above, regression from moderate albuminuria to normal AER was noted in 35% of the study participants, but more than half of the regressors relapsed to persistent moderate albuminuria when the follow-up was closed (15). Albuminuria re-appeared in every fourth individual who had regressed to normal AER in the DCCT trial (38), as well as in the Oxford regional prospective study (16). In one study, the 5-year relapse rate from regressed normal AER back to moderate albuminuria was as high as 100% (18).

## The association between albuminuria regression and clinical factors

Large type 2 diabetes trials, such as the RENAAL (Reduction of Endpoints in NIDDM with the Angiotensin II Antagonist Losartan) (58), the IDNT (Irbesartan in Diabetic Nephropathy Trial) (59), and the MARVAL (MicroAlbuminuria Reduction With VALsartan) Study (60) have linked the use of renin-angiotensin system (RAS) blockers to higher chances of regression of albuminuria compared to placebo. Similarly, the EMPA-REG OUTCOME trial (empagliflozin) (61), CANVAS Program (62), and CREDENCE trial (canagliflozin) (63) have shown significant rates of albuminuria regression following the initiation of SGLT2 (sodium-glucose cotransporter-2) inhibitor use. In the context of type 1 diabetes, data on SGLT2 inhibitor use are unavailable, and the evidence of antihypertensive medication is less conclusive.

Among those demonstrating beneficial effects on albuminuria after intensified hypertension treatment, the Captopril Trial has played a pivotal role (64). This landmark trial, conducted by the Collaborative Study Group in the early 1990s, revealed significant reductions in albuminuria, and an increased likelihood of remission from nephrotic-range albuminuria (65), when individuals with type 1 diabetes were treated with the ACE (angiotensin-converting enzyme) inhibitor captopril. Building upon the findings of the Captopril Trial, a randomized controlled trial conducted by The European Microalbuminuria Captopril Study Group in 1994 further solidified the evidence by revealing considerable reductions in albuminuria for captopril treatment (66). A meta-analysis from 2001, spanning 12 studies with normotensive individuals with type 1 diabetes and moderate albuminuria, landed at an odds ratio of 3.07 for regression to normal AER in the ACE-inihibtor arm vs. placebo/nonintervention group (67). However, the confidence intervals in most of the included studies did not exclude unity.

In fact, many studies have failed to establish a consistent association between antihypertensive medication and regression of albuminuria and instead noted high rates of spontaneous regression. For instance, in the groundbreaking study from the Joslin Center, the use of ACE inhibitors was not associated with regression (30). As the treatment guidelines encourage RAS blocker initiation when moderate albuminuria appears, findings from observational studies may be distorted by high prevalence of treatment in both the regression and the non-regression groups. Nevertheless, although it is ambiguous whether RAS blocker use induces albuminuria regression in type 1 diabetes, optimized arterial blood pressure has repeatedly been associated with albuminuria regression in the patient group (15, 30), as has salutary glycemic control (29, 30). Other suggested factors behind regression are largely the same that have appeared as risk factors for albuminuria progression (yet with an inverse direction); such as lower serum cholesterol and triglycerides (15, 30, 31), sex (regression more likely in women) (29), lower initial AER (15, 31), and short duration of moderate albuminuria (30). Regression has also been linked to lower KIM-1 and NAG, that is, markers of tubular dysfunction, as compared to those undergoing albuminuria progression (35). In addition, a positive correlation between the likelihood of regression and insulin sensitivity has been found in the CACTI cohort (36).

## The propitious clinical consequences of albuminuria regression

**Figure 2:**
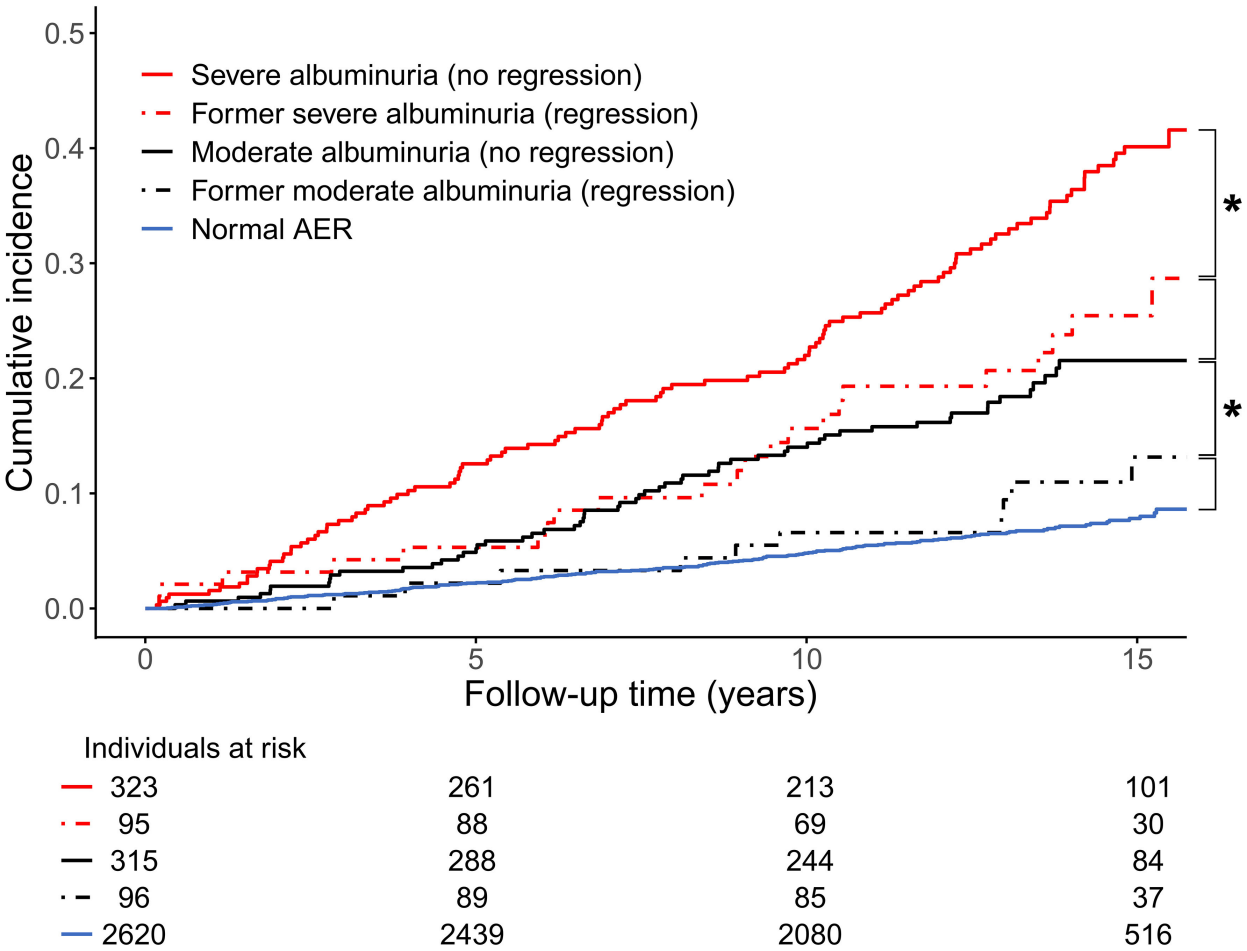
Cumulative incidence of cardiovascular disease event (myocardial infarction, coronary procedure, or stroke) stratified by baseline status of albuminuria in the Finnish Diabetic Nephropathy (FinnDiane) Study. * denotes p<0.05 between groups. AER, albumin excretion rate. Adapted from (37) with permission from Springer Nature.

Studies on the clinical consequences of albuminuria regression in type 1 diabetes have also given rise to conflicting results. As reviewed earlier, it is widely recognized that albuminuria is a marker of increased risk of cardiovascular disease and premature mortality, and that the risk increases with advancing stage of kidney disease. However, it is more complex whether and to what extent diminishing AER to a less advanced stage will indicate a change in the risk of other vascular endpoints. Notably, in type 2 diabetes, beneficial kidney and cardiovascular effects have been observed after achieving regression of albuminuria (68–70). In type 1 diabetes, two studies with opposing views on the subject have been published:

In the Finnish Diabetic Nephropathy (FinnDiane) study cohort, the course of albuminuria was assessed among 913 individuals with initial moderate (N=438) and severe (N=475) albuminuria. Interestingly, independently of the initial albuminuria stage, the regression rate was 23%. The status of regression, defined as a change to a less advanced category of albuminuria, was categorized at the study baseline, and the occurrence of a combined cardiovascular endpoint and premature mortality was evaluated during a median follow-up of 14 years. The clinical findings were optimistic: Regression of albuminuria, both from the severe and moderate stage to a less advanced one, conferred a risk reduction of adverse CVD events to the same level as for those whose DKD stage did not progress in the first place (**Figure 2**). The same was true for premature mortality (37).

However, findings from the DCCT/EDIC did not align with those from the FinnDiane cohort: After study initiation, 423 participants developed moderate albuminuria and 171 of these regressed – transiently or permanently – back to normal AER. The DCCT/EDIC participants were followed for a median of 15.7 years after the regression had occurred. During this follow-up, the risk of a composite cardiovascular disease event in the regressed group was significantly higher – 2.62-fold – than those with consistent normal AER. Regression of albuminuria was neither associated with a reduced cardiovascular risk in comparison with the sustained moderate albuminuria category (hazard ratio 1.33 [95% confidence interval 0.68-2.59]) (38).

The discrepancy is unexpected because of the many similarities in the two studies, such as the comparable cohort sizes and analogous endpoint definitions. The follow-up was somewhat longer in the DCCT/EDIC; however, between-group differences appeared early in the prospective phase of the FinnDiane, so this is neither a plausible explanation. On the other hand, the difference may stem – for instance – from different cohort characteristics (the FinnDiane Study participants were older, had a longer diabetes duration, and had higher blood pressure at baseline). In addition, the studies represent very different designs, namely a multicenter observational study (FinnDiane) vs. an intervention study (the DCCT). Nevertheless, updated information from other large cohorts is needed before definite conclusions on whether albuminuria regression indicates a change in the vascular risk profile or not can be drawn.

## Kidney failure in type 1 diabetes

Register-based research has played a crucial role in untangling the occurrence of kidney failure, depicted by a need for maintenance dialysis or a kidney transplant for survival, among individuals with type 1 diabetes. A few studies on temporal trends have also been published. Notably, with some exceptions, available investigations almost exclusively point towards a decline in the incidence rates over time.

A noteworthy Swedish nationwide and population-based study conducted in 2010 focused on individuals with type 1 diabetes onset in 1977 or later, encompassing a substantial sample size of 11,681 (39). The study found a 30-year cumulative incidence of kidney failure at 3.3%. This proportion was lower than what had been published earlier; however, the results showed no effect of a so-called calendar effect within the cohort, in contrast to many others.

Similar cumulative incidence rates have since been observed in three other influential studies. A Japanese single-center study led by Otani et al. revealed a 25-year cumulative incidence of 3.0% among those diagnosed with type 1 diabetes between 1985 and 1999 (40). For individuals with an earlier diabetes onset (1961 to 1984), the corresponding proportion was notably higher, 11.7%. In a population-based Norwegian study, which considered individuals diagnosed with type 1 diabetes during two distinct time frames (1973-82 and 1989-2012), the 30-year cumulative incidence was 2.9% (41). Interestingly, there was no significant difference in incidence between the two diagnosis cohorts.

Furthermore, a comprehensive nationwide population-based Finnish study, which encompassed virtually all individuals diagnosed with type 1 diabetes between 1965 and 2011, showed a 30-year cumulative incidence of 4.4% for those diagnosed after 1980 (42). The cumulative incidence was higher at 7.0% when considering all individuals across the study period. In contrast to the Swedish (39) and Norwegian (41) studies, there was a consistent decrease in cumulative incidence over time. Notably, the decline was particularly pronounced between the 1965-1969 cohort and subsequent diagnosis cohorts. This attribute likely accounts for the discrepancy between this and the two studies above, as the populations are in many aspects considered similar (due to their geographic proximity and historical interactions), with matching recommendations and accessibility to diabetes care – thus, unlikely to explain the dissimilarity.

In addition to those above, data from the United States support a declining trend of kidney failure in type 1 diabetes; however, with substantially substantially higher cumulative incidences (especially at earlier calendar year at diabetes onset) than in the other recent reports from Europe and Japan. In the Pittsburgh EDC study, among individuals with diabetes onset in 1950-64, the 30-year cumulative incidence of kidney failure was as high as 34.6%, the 40-year cumulative incidence 48.5%, and the 50-year cumulative incidence 61.3% (17). These were substantially greater than, for instance, in the Joslin Center, where the 35-year cumulative incidence was around 20% among individuals with diabetes since the late 1950s (71). Fortunately, a dramatic reduction in the kidney failure burden in the Pittsburgh EDC study was noted over time, as the 30-year cumulative incidence had fallen to 14.5% and 40-year to 26.5% in those with diabetes debut in 1965-80 (p for between-group difference <0.001). Nevertheless, considering the grim consequences of diabetic kidney disease – especially at the advanced disease stages – these proportions remain alarmingly high. Importantly, geographical differences in the epidemiology of diabetic kidney disease warrant further consideration.

## Discussion

Diabetic kidney disease remains a significant burden for individuals with type 1 diabetes. In modern times, it affects one in three individuals (at the stage of moderate albuminuria), and 3-4% of those with type 1 diabetes require kidney replacement therapy within 30 years after their diabetes diagnosis. The disease progression comes with grim cardiovascular consequences. Unfortunately, after an initial significant reduction during the mid-20th century, the incidences of moderate and severe albuminuria have not fallen after the 1980s. Reasons behind this can be speculated upon. First, as insulin resistance has repeatedly been associated with increased diabetic kidney disease risk, the rising prevalence of obesity – also within the type 1 diabetes population – may explain why the albuminuria incidence has not dropped, although hyperglycemia treatment has improved. Another vital hypothesis is the lack of efficient kidney-protective treatment approaches during the past 40 years. In the early 1980s, aggressive blood pressure treatment was found to slow kidney function deterioration among individuals with type 1 diabetes (72, 73), and subsequent findings a few years later proved the superiority of ACE inhibitors over other antihypertensive drugs (74–76). However, after this, the progress has been limited. The situation is the contrary in type 2 diabetes, where last years’ groundbreaking discoveries related to SGLT2 inhibitors have undoubtedly enhanced the overall prognosis for this group of individuals. However, the medication class has not been approved for type 1 diabetes due to the increased risk of ketoacidosis. Although off-label use of this drug class for precisely chosen individuals with type 1 diabetes occurs, it is noteworthy that the greatest risk of ketoacidosis has been observed in those with far-advanced kidney disease (77). In other words, the group with the most urgent need of kidney-protective adjunctive medication is also the group with the highest ketoacidosis risk. Another drug of particular interest is finerenone, a non-steroidal mineralocorticoid receptor antagonist with beneficial kidney- and cardiovascular effects in type 2 diabetes. The first finerenone trial to include individuals with type 1 diabetes has recently been initiated (78), so findings during the upcoming years will tell whether this drug class will be suitable for type 1 diabetes as well.

As albuminuria regression has, in some reports, been shown to be associated with an improved overall prognosis, the methods to achieve it – and to avoid progression to kidney disease in the first place – need a further breakdown. Further research will eventually tell whether non-steroidal mineralocorticoid receptor antagonists, SGLT2 inhibitors, or some other type of treatment with kidney benefits will be suitable for type 1 diabetes. Until updated evidence is available, it is crucial that timely albuminuria screening and efficient glycemia, blood pressure, and lipid control are not overlooked in individuals with type 1 diabetes, to minimize the vicious consequences of diabetic kidney disease.

## Author contributions

FJS: Conceptualization, Funding acquisition, Writing – original draft, Data curation. P-HG: Conceptualization, Funding acquisition, Supervision, Writing – review & editing.
